# Repeatability of Quantitative Sodium Magnetic Resonance Imaging for Estimating Pseudo-Intracellular Sodium Concentration and Pseudo-Extracellular Volume Fraction in Brain at 3 T

**DOI:** 10.1371/journal.pone.0118692

**Published:** 2015-03-09

**Authors:** Guillaume Madelin, James Babb, Ding Xia, Ravinder R. Regatte

**Affiliations:** Center for Biomedical Imaging, Department of Radiology, New York University Langone Medical Center, New York, NY, USA

## Abstract

The purpose of this study is to assess the repeatability of the quantification of pseudo-intracellular sodium concentration (C_1_) and pseudo-extracellular volume fraction (α) estimated in brain in vivo using sodium magnetic resonance (MRI) at 3 T. Eleven healthy subjects were scanned twice, with two sodium MRI acquisitions (with and without fluid suppression by inversion recovery), and two double inversion recovery (DIR) proton MRI. DIR MRIs were used to create masks of gray and white matter (GM, WM), that were subsequently applied to the C_1_ and α maps calculated from sodium MRI and a tissue three-compartment model, in order to measure the distributions of these two parameters in GM, WM or full brain (GM+WM) separately. The mean, median, mode, standard deviation (std), skewness and kurtosis of the C_1_ and α distributions in whole GM, WM and full brain were calculated for each subject, averaged over all data, and used as parameters for the repeatability assessment. The coefficient of variation (CV) was calculated as a measure of reliability for the detection of intra-subject changes in C_1_ and αfor each parameter, while intraclass correlation (ICC) was used as a measure of repeatability. It was found that the CV of most of the parameters was around 10–20% (except for C_1_ kurtosis which is about 40%) for C_1_ and α measurements, and that ICC was moderate to very good (0.4 to 0.9) for C_1_ parameters and for some of the α parameters (mainly skewness and kurtosis). In conclusion, the proposed method could allow to reliably detect changes of 50% and above of the different measurement parameters of C_1_ and αin neuropathologies (multiple sclerosis, tumor, stroke, Alzheimer’s disease) compared to healthy subjects, and that skewness and kurtosis of the distributions of C_1_ and αseem to be the more sensitive parameters to these changes.

## Introduction

Sodium ions (^23^Na^+^) are vital components in the human brain, and their homeostasis is a major process in cells through coupled exchange with potassium ions K^+^ between the intra- and extracellular compartments through the Na^+^/K^+^-ATPase (sodium-potassium pump) [1]. This pumping process maintains a constant gradient of sodium concentration across the cell membrane (about 10–15 mM intracellular versus 140 mM extracellular), which is used to control cell volume, pH balance, glucose and neurotransmitter transport, membrane electrical potential (and pulse transmission), and protect the cells from swelling. Dysregulation of the sodium-potassium pump, or of ATP-dependent processes in the cells, will provoke dysregulation of ion homeostasis and therefore leads to an increase of intracellular sodium concentration (C_1_) as the gradient cannot be sustained anymore, and furthermore to cell death and subsequent increase of extracellular volume fraction (*α*). These two parameters (C_1_ and *α*) are very sensitive to cell viability and ion homeostasis [2–4]. Measuring variations in C_1_ in brain in vivo could help assess the degree of cell hypometabolism or injury [3], tumor malignancy [5, 6] or resistance to therapy [7]. Measuring variations in *α* could give more information on effusion or disruption of cell packing [8], dehydration [9], changes in vascularization and tumor edema angiogenesis [10, 11] or metabolite clearance in the brain [12]. Measuring both C_1_ and *α* in vivo could therefore be of great importance for assessing early signs of neuropathologies characterized by a loss of cell integrity or homeostasis, such as brain tumors [13–15], multiple sclerosis [16], stroke [17, 18], or Alzheimer’s disease [19].

Sodium magnetic resonance imaging (MRI) [2, 20, 21] is a non-invasive MRI technique based on the detection of the sodium ions present in different concentrations in biological tissues [2, 20, 22], that could allow us to measure directly C_1_ and *α* in a quantitative manner. We recently developed a simple method based on sodium MRI along with double inversion recovery (DIR) proton MRI, for estimating these two parameters in the gray matter (GM), white matter (WM) and full brain separately [23]. This method is based on two sodium acquisitions, with and without fluid (cerebro-spinal fluid—CSF—and extracellular) suppression by inversion recovery, and a three-compartment model (intracellular, extracellular and solid compartments) for quantifying simultaneously C_1_ and *α* in brain. In this article, we will refer to C_1_ and *α* as the pseudo-intracellular sodium concentration and pseudo-extracellular volume fraction, respectively. The term ‘pseudo’ represents experimental uncertainties arising from low signal-to-noise ratio (SNR) of sodium MRI, partial volume effects, inter-compartmental T1 variations (between intracellular and extracellular spaces), imperfect inversion pulse, and presence of signal from bound sodium in the extracellular compartment that is not completely suppressed by IR and which can therefore reduce the accuracy of C_1_ and *α* calculations. The final results can be presented as 3D maps of C_1_ and *α* and as distributions of C_1_ and *α* values for whole GM, WM or full brain. These distributions can be characterized by global statistical measures such as mean, median, mode, standard deviation (std), skewness or kurtosis, which could be used for detection of pathologies in brain. In the present work, we want to assess the repeatability of these measures on healthy volunteers scanned twice (within a month, on average) on a clinical 3 T scanner, in order to determine the order of magnitude of the changes in C_1_ and *α* that could be detectable in patients with neuropathologies.

## Materials and Methods

### Volunteers and Ethics Statement

The brains of eleven healthy volunteers (6 men, 5 women, mean age = 32.1 ± 7.9 years) were scanned twice within a month (on average) with exactly the same proton/sodium protocol. This study was approved by the institutional review board (IRB) of New York University Langone Medical Center and all volunteers signed informed consent form (#11783, Technical Development of up to 7T Magnetic Field Level) prior to the scans.

### MRI hardware

All scans were performed at 3 T on a Tim Trio system (Siemens, Erlangen, Germany) using a dual-tuned ^1^H/^23^Na birdcage radiofrequency (RF) coil tuned at 128/33 MHz (Stark Contrast, Erlangen, Germany).

### Proton MRI acquisition

Two double inversion recovery (DIR) MRI acquisitions were performed. The first DIR image was acquired in order to suppress both CSF and WM using the DIR Turbo Spin Echo SPACE sequence [24, 25] with the following parameters: TR = 7500 ms, TE = 300 ms, field-of-view (FOV) = 220×320×320 mm^3^ isocenter, resolution = 2.5 mm isotropic, inversion times TI_1_ = 2650 ms and TI_2_ = 550 ms, time of acquisition (TA) = 4:00 min. The second DIR image was acquired in order to suppress both CSF and GM with the same parameters as the first DIR except TI_1_ = 2800 ms and TI_2_ = 800 ms.

### Sodium MRI acquisition

Sodium acquisitions were performed using the 3D ultrashort echo time (UTE) non-Cartesian FLORET sequence [26] with the following parameters:

1. Sequence 1—without fluid suppression: TR = 80 ms, TE = 0.2 ms, flip angle (FA) = 80°/0.5 ms, 3 hubs at 45°, 200 interleaves/hub, 745 data points / interleaf, dwell time 10 *μ*s, 14 averages, FOV = 320 mm isotropic, acquisition resolution = 5 mm isotropic. TA = 11:00 min.
2. Sequence 2—with fluid suppression by inversion recovery (IR): a ‘soft’ rectangular inversion pulse [27] of 180°/6 ms was added to the FLORET sequence with an inversion time TI = 24 ms (calculated from the centers of the pulses), TR = 100 ms, TE = 0.2 ms, FA = 90°/0.5 ms, 3 hubs at 45°, 85 interleaves/hub, 746 data points / interleaf, dwell time 10 *μ*s, 40 averages, FOV = 320 mm isotropic isocenter, acquisition resolution = 6.7 mm isotropic. TA = 17:00 min. A spoiler gradient of 4 ms was also included during TI for removing any transverse magnetization generated by imperfections of the inversion pulse.

All sodium images were reconstructed offline in Matlab (MathWorks, Natick, MA, USA) with standard 3D regridding [28] and density compensation [29] with a nominal resolution of 2.5 mm isotropic (128×128×128 voxels), matching the nominal resolution of the DIR proton images.

### Data processing summary

**Fig 1:**
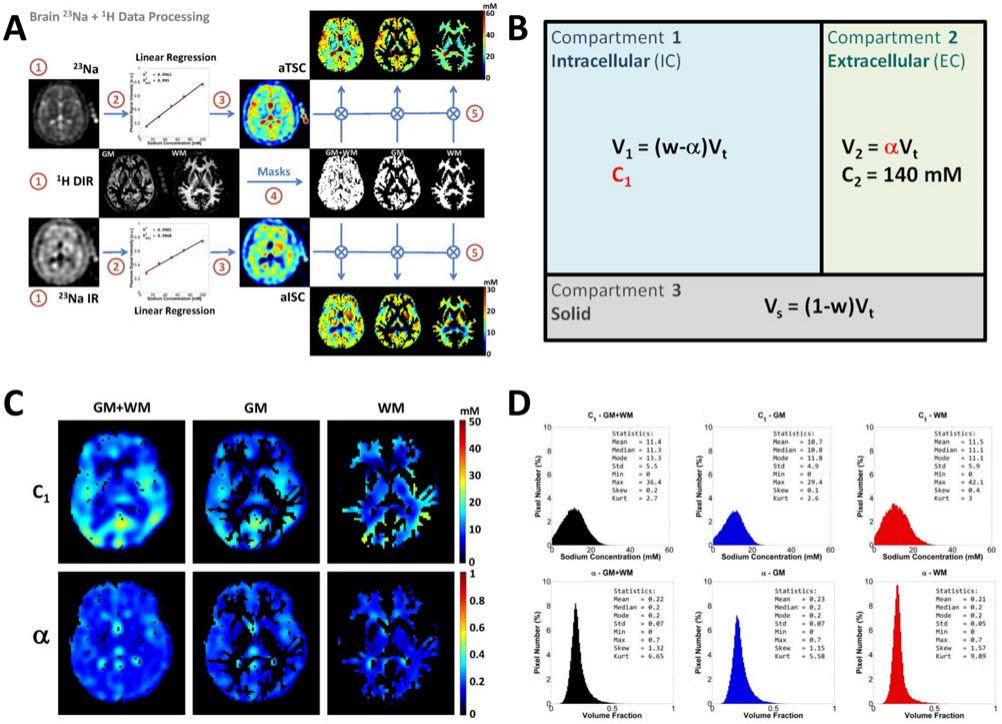
Summary of the brain data MRI acquisition and processing. **A**. (**1**) Sodium MRI acquisition with and without IR, and proton DIR acquisition. (**2**) Linear regression of reference phantoms signals. (**3**) Calculation of aTSC and aISC maps. (**4**) Calculation of the masks of GM, WM and full brain (GM+WM) from DIR data. (**5**) Multiplication of sodium maps by the masks. **B**. Three-compartment model: compartment 1 = intracellular space of volume V_1_ and sodium concentration C_1_; compartment 2 = extracellular space of volume V_2_, sodium concentration C_2_ and extracellular volume fraction *α*; compartment 3 = solid compartment of volume V_*s*_ and no sodium. Total volume is V_*t*_ = V_1_+ V_2_ +V_*s*_. Water volume fraction is denoted w. **C**. C_1_ and *α* maps in full brain, GM, and WM calculated from aTSC, aISC and the 3-compartment model. **D**. Histograms of C_1_ and *α* values over the whole full brain, whole GM and whole WM data. GM = gray matter, WM = white matter, GM+WM = full brain. Reproduced from Figs. 1–4 from Ref. [23].

The data processing (in Matlab) for calculating the final pseudo-intracellular sodium concentration (C_1_) and pseudo-extracellular volume fraction (*α*) maps is described in details in Ref. [23]. In the present article, we will just summarize this data processing in 4 steps:

1. **Calibration phantoms** **and linear regression**: The signal from five calibration phantoms (Agar gel 3% with 10, 30, 50, 70 and 100 mM NaCl, 17 mm diameter and 100 mm length) placed within the FOV on the side of the head was measured and averaged over 4 consecutive slices (10 voxels/phantom/slice). Their relaxation times were also measured as T1 = 38 ms and T2* = 7 ms at 3 T. The loss of signal of the sodium phantoms due to relaxation during RF pulses and delays was estimated by full density operator simulation of spin 3/2 dynamics [30–32] during the RF pulse sequence and was used to correct the phantom signals, by factors the 1.10 (sequence 1) and 1.60 (sequence 2). Linear regression of the corrected signals of the phantoms versus their sodium concentrations was then performed (Fig. 1A). Linear regression was considered as valid only when the coefficients of determination R^2^ ⩾ 0.99 and adjusted $R_{adj}^{2}⩾0.98$ [33], in order to improve the robustness of the method against noise and signal variations in the phantoms.
2. **Intermediate sodium maps**: The apparent total sodium concentration (aTSC) and apparent pseudo-intracellular sodium concentration (aISC) maps were calculated from sequences 1 and 2, respectively, using the coefficients of the linear regression of phantom signals (Fig. 1A). Using average sodium relaxation times in brain from the literature [2, 14, 20, 21, 27] (T1∼35 ms, and T2_*short*_∼5 ms, T2_*long*_∼25 ms, in parenchyma), correction factors of the sodium maps (0.85 for aTSC and 0.5 for aISC) were calculated using full density operator simulation of the sodium spin dynamics [30–32] during the RF pulse sequences 1 and 2 respectively.
3. **GM and WM masks**: 3D GM and WM and full brain (WM+GM) masks were calculated from the ^1^H DIR acquisitions using SPM8 [34] in Matlab. From the resulting GM (or WM) SPM probability map, only pixels with a probability ⩾ 0.75 of being in the GM (or WM) were kept in the GM (or WM) mask. The aTSC and aISC maps were then multiplied by the GM, WM and full brain masks (Fig. 1A). These masked aTSC and aISC maps were therefore used for the quantification of C_1_ and ***α*** in order to measure the distributions of these 2 parameters separately in WM, GM and full brain.
4. **C_1_ and *α* maps**: Pseudo-intracellular sodium concentration (C_1_) and pseudo-extracellular volume fraction (*α*) quantification was based on a simple three-compartment model (Fig. 1B). In this model, the extracellular compartment (including interstitial volume, CSF, plasma and blood) has a constant average sodium concentration C_2_∼140 mM [2, 18, 20, 21, 35]. We also considered that the water (fluid) volume fraction is constant and take averages values w_*WM*_ = 0.7, w_*GM*_ = 0.85 and w_*brain*_ = 0.775 (mean value from WM and GM)[36–38]. We also assumed here that all extracellular sodium signals are completely suppressed by inversion recovery in sequence 2, or within noise level of the image (due to imperfection of inversion pulse and possible variations of T1 from CSF and other extracellular fluids). The value of each voxel of the aTSC map is by definition equal to the total sodium concentration within each voxel: aTSC = (C_1_×V_1_+C_2_×V_2_)/V_*t*_ (with V_*t*_ = total volume of the voxel). The value of each voxel of the aISC map is by definition equal to the intracellular sodium concentration only: aISC=(C_1_×V_1_)/V_*t*_. From these assumptions and equations, we can calculate the unknown parameters C_1_ and *α* of interest, using the relationships given in Fig. 1B and with w taking the values w_*WM*_, w_*GM*_ and w_*brain*_ depending on the masked aTSC and aISC maps used: α = (aTSC−aISC)/C_2_ and C_1_ = (C_2_×aISC)/(w×C_2_−aTSC+aISC). This calculation is performed for each voxel. All voxels are then recombined in maps of C_1_ and *α* in WM, GM and full brain, as shown in Fig. 1C. The distribution of values of C_1_ and *α* over whole WM, GM or full brain can also be plotted as histograms (Fig. 1D), and the properties of each distribution can be characterized for each volunteer in terms of histogram summary statistics: mean, median, mode, standard deviation (std), skewness and kurtosis. The reliability of the method will be assessed in terms of repeatability of these six summary statistics.

Note that no RF (B_1_) map was acquired in this preliminary study, as it was not deemed necessary from preliminary data acquired while testing this technique. We used a birdcage coil with homogeneous transmit and receive B_1_ fields, no improvement in the quality of the images, nor in the sodium data quantification was observed when B_1_ correction was applied. Moreover, B_1_ correction for the fluid suppressed images (from inversion recovery) is not trivial, as the effect of B_1_ inhomogeneities on the images depends not only on the pulses (inversion and excitation) but also on the T1 of the tissues and the relaxation of their magnetization during the inversion time, which varies in different compartments of the brain.

### Lesion simulations

Simulations of ‘fluid’ (cystic-like) and ‘solid’ (tumor-like) lesions, either compact (10×10×10 voxels) or randomly distributed over the whole brain (1000 voxels over 86571, which correspond to about 1.15% of all voxels in brain) were also performed and are presented in Supporting Information (S1 File). These simulations are similar to the ones presented in Ref. [23] Supplementary Material. These simulated lesions include voxels with partial volume effect from the CSF. Random 1-voxel lesions are distributed over the whole brain (including voxels with partial volume effect from CSF). Details are given in the captions of the figures A to D in S1 File.

### Statistical analysis

Restricted maximum likelihood estimation of the variance components in a random effects model was used to estimate the intra-subject variance (i.e., the variance between results from replicate scans of the same subject) and inter-subject variance (the variance between results from different subjects) of each measure (i.e., mean, median, mode, std, skewness and kurtosis) within each tissue. The estimated variance components were used to compute the coefficient of variance (CV, in %) as the square root of the intra-subject variance expressed as a percentage of the mean, and the intra-class correlation (ICC) as the inter-subject variance divided by the sum of the intra- and inter-subject variances. By expressing the intra-subject variance relative to the overall mean of a given measure, the CV is considered an indicator of the utility of a measure for detecting within-subject changes over time; temporal changes that are not substantively greater in magnitude than the CV would be difficult to distinguish from changes attributable to random noise. It is noted that the CV is not appropriate for measures (e.g., skewness) that can be both positive and negative since the mean of such a measure does not represent the magnitude of a typical observed value of the measure. The ICC is a measure of the repeatability (or test-retest reliability) of the method [39]. It ranges from 0 to 1, with values near 1 implying that the ratio of the intra-subject variance to the inter-subject variance is close to 0. As a result, the ICC should be high (close to 1) if a measure is to be potentially useful for the detection of a mean difference between subject groups. In a similar fashion as described in Ref. [40], repeatability was regarded as very good if ICC ⩾ 0.8 (•• symbol in the tables), good if 0.6 ⩽ ICC < 0.8 (• symbol), fair/moderate if 0.4 ⩽ ICC < 0.6 (∘ symbol) and poor if ICC < 0.4 (◊ symbol). Similarly, CV was regarded as very good if CV ⩽ 10% (•• symbol in the tables), good if 10% < CV ⩽ 20% (• symbol), fair/moderate if 20% < ICC ⩽ 30% (∘ symbol) and poor if CV > 30% (◊ symbol). Statistical computations were performed with SAS 9.3 (SAS Institute, Cary, NC).

## Results

**Fig 2:**
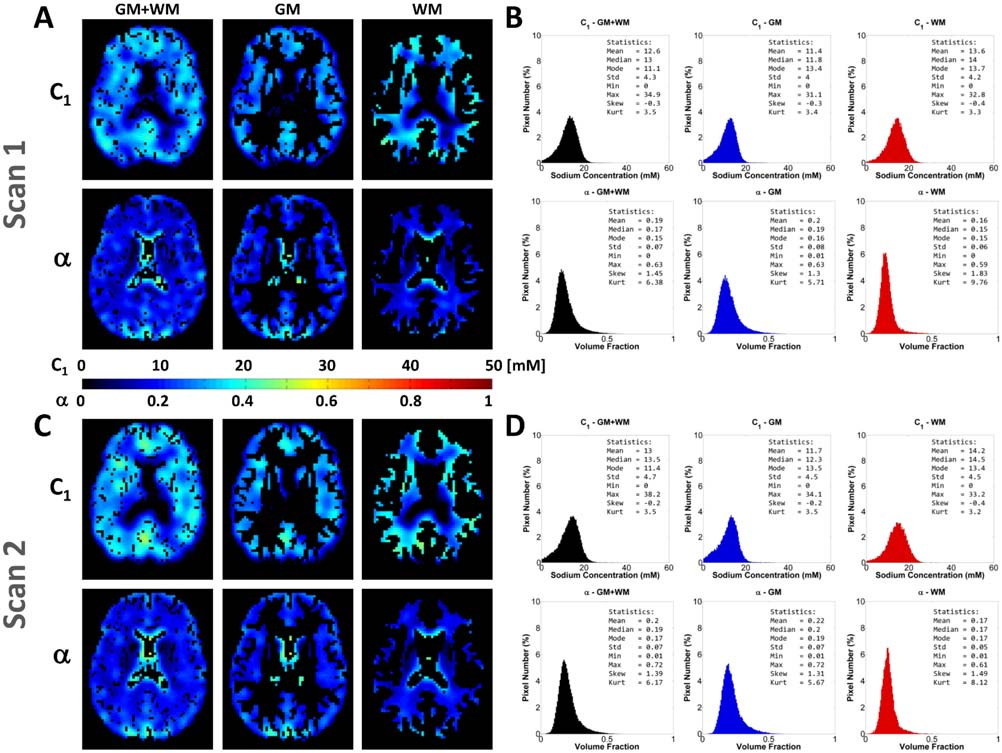
Examples of scan-rescan C_1_ and *α* maps (1 transverse slice) and distributions (over whole GM, WM and full brain) for one volunteer. **A**. Scan 1: C_1_ and *α* maps. **B**. Scan 1: C_1_ and *α* distributions. **C**. Scan 2: C_1_ and *α* maps. **D**. Scan 2: C_1_ and *α* distributions. Transverse slices were matched visually as well as possible.

**Fig 3:**
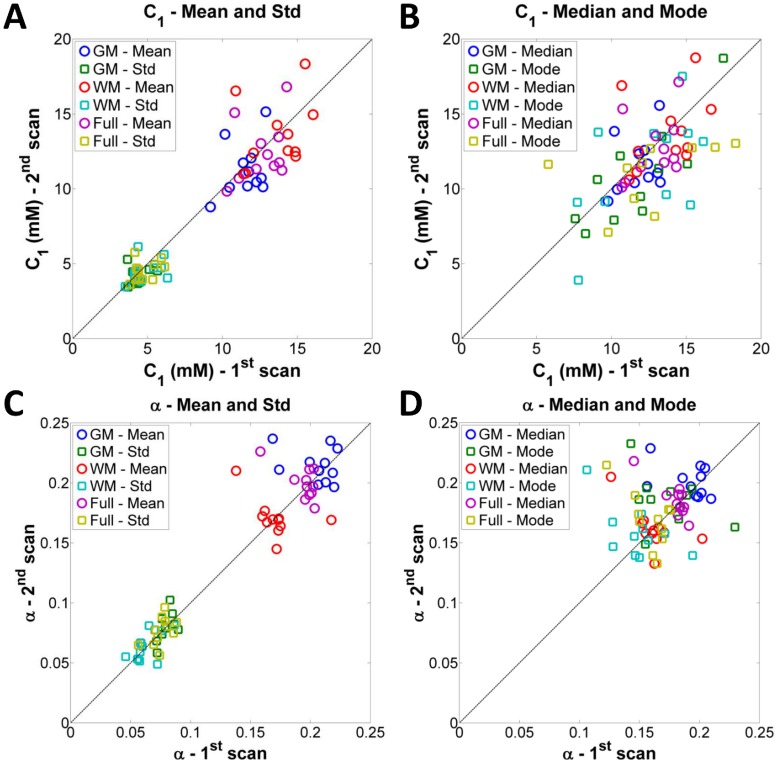
Plots of mean, median, mode and standard deviation (std) of the C_1_ and *α* distributions from 11 volunteers scanned twice (1^st^ scan vs. 2^nd^ scan). **A**. Mean and std of C_1_. **B**. Median and mode of C_1_. **C**. Mean and std of *α*. **D**. Median and mode of *α*. GM = gray matter, WM = white matter, Full = full brain (GM+WM).

**Fig 4:**
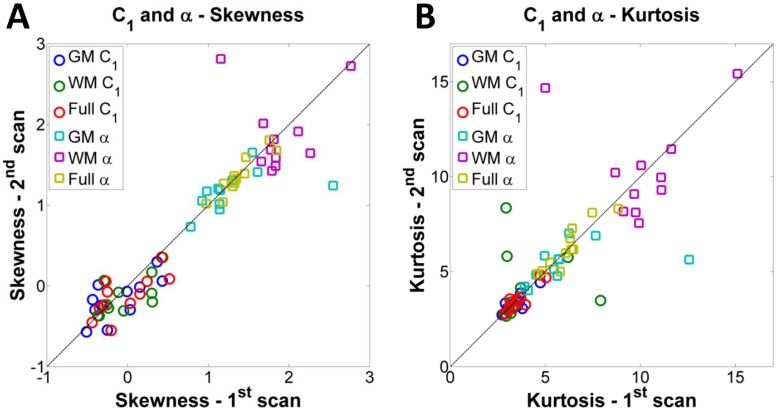
Plots of skewness and kurtosis of the C_1_ and *α* distributions from 11 volunteers scanned twice (1^st^ scan vs. 2^nd^ scan). **A**. Skewness of C_1_ and *α*. **B**. Kurtosis of C_1_ and *α*. GM = gray matter, WM = white matter, Full = full brain (GM+WM).

Fig. 2 show an example C_1_ and *α* maps and distributions from two scans of the same volunteer. Note the higher intensities, mainly in *α* maps, near the ventricles, due to residual partial volume effect from CSF. The C_1_ and *α* distributions from scan and re-scan present very similar shapes between the two scans, for GM, WM and full brain (GM+WM). Fig. 3 and 4 show scatter plots of mean, median, mode, std, skewness and kurtosis of C_1_ and *α* from the eleven volunteers scanned twice (1^st^ scan vs. 2^nd^ scan), in GM, WM and full brain. Data from the histogram statistics for all volunteers and all scans can be seen in Supporting Information (S1 Dataset). On Fig. 3, we observe that all mean, median and mode C_1_ values from scan/re-scan are in the range 5–20 mM. The mean, median and mode *α* values are in the range 0.10–0.25, over whole GM, WM and full brain. Std C_1_ values are all regrouped around 5 mM, while std *α* values are in the range 0.05–0.10. On Fig. 4, we observe that most of the values of skewness and kurtosis are close to the diagonal (good match between scan and re-scan measurements), with the exception of a few data points. Skewness values for C_1_ are all regrouped around 0, while they are in the range 1–3 for *α*. Kurtosis values for C_1_ are tightly regrouped in the range 3–5, while they are more spread out for *α*, in the range 5–15.

Tables 1 and 2 present the mean_all_, std_all_, CV and ICC of the C_1_ and *α* measurements, over all data (n = 22, from 11 volunteers scanned twice). Note that the mean and std over all data were labeled mean_all_ and std_all_ to avoid confusion with the mean and std of the measurements of C_1_ and *α* for each volunteer and scan. Results can be summarized as below.

**Table 1:** Repeatability of pseudo-intracellular sodium concentration (C_1_).

**C_1_ (mM)**	**Mean_all_**	**Std_all_**	**Inter-Var**	**Intra-Var**	**CV (%)**		**ICC**	
**GM**								
Mean	**11.41**	1.49	1.48	1.53	10.8	•	0.492	∘
Median	**11.74**	1.55	1.60	1.66	11.0	•	0.491	∘
Mode	**11.26**	3.11	9.02	2.13	13.0	•	0.809	••
Std	**4.32**	0.64	0.28	0.27	11.9	•	0.517	∘
Skewness	**-0.14**	0.29	0.07	0.03	NA		0.732	•
Kurtosis	**3.37**	0.51	0.26	0.05	6.4	••	0.846	••
**WM**								
Mean	**13.59**	2.02	2.80	2.67	12.0	•	0.511	∘
Median	**13.71**	2.20	3.37	3.10	12.8	•	0.520	∘
Mode	**11.89**	3.39	9.18	5.23	19.2	•	0.637	•
Std	**4.66**	0.87	0.51	0.52	15.5	•	0.493	∘
Skewness	**-0.07**	0.27	0.06	0.03	NA		0.681	•
Kurtosis	**4.01**	1.68	1.51	2.62	40.4	◊	0.365	◊
**Full brain**								
Mean	**12.56**	1.69	1.98	1.90	11.0	•	0.510	∘
Median	**12.79**	1.80	2.26	2.09	11.3	•	0.520	∘
Mode	**12.31**	3.41	7.89	7.85	22.8	∘	0.501	∘
Std	**4.66**	0.73	0.36	0.36	12.9	•	0.497	∘
Skewness	**-0.08**	0.29	0.07	0.03	NA		0.728	•
Kurtosis	**3.58**	0.66	0.44	0.04	5.6	••	0.916	••

Mean and standard deviation (two first columns) of mean, median, mode, standard deviation (std), skewness and kurtosis of C_1_ were calculated over all data (n = 22, from 11 volunteers scanned twice). Note that the mean and std over all data were labeled mean_all_ and std_all_ to avoid confusion with the mean and std of the measurements of C_1_ and *α* for each volunteer and scan. Inter- and intra-subject variance (Inter-Var and Intra-Var respectively), coefficient of variation (CV, in %) and intra-class correlation (ICC) were calculated as described in the Statistical analysis section (see text). NA: Not available; the CV is not appropriate for measures (e.g., skewness) that are not inherently non-negative. Symbols represent subjective evaluation of CV and ICC: •• = very good (ICC ⩾ 0.8 or CV ⩽ 10%), • = good (0.6 ⩽ ICC < 0.8 or 10% < CV ⩽ 20%), ∘ = fair/moderate (0.4 ⩽ ICC < 0.6 or 20% < CV ⩽ 30%), ◊ = poor (ICC < 0.4 or CV ⩾ 30%).

**Table 2:** Repeatability of pseudo-extracellular volume fraction (*α*).

***α***	**Mean_all_**	**Std_all_**	**Inter-Var**	**Intra-Var**	**CV (%)**		**ICC**	
**GM**								
Mean	**0.210**	0.017	0.0001	0.0003	8.9	••	0.217	◊
Median	**0.196**	0.016	0.00007	0.0004	9.7	••	0.169	◊
Mode	**0.180**	0.235	0.0002	0.0007	15.3	•	0.181	◊
Std	**0.078**	0.011	0.00009	0.00005	8.8	••	0.657	•
Skewness	**1.24**	0.38	0.10	0.08	NA		0.549	∘
Kurtosis	**5.74**	1.82	2.19	2.32	26.6	∘	0.485	∘
**WM**								
Mean	**0.171**	0.017	0.00007	0.0004	11.8	•	0.155	◊
Median	**0.161**	0.017	0.00005	0.0005	13.3	•	0.104	◊
Mode	**0.153**	0.022	0.0001	0.0008	18.1	•	0.108	◊
Std	**0.063**	0.011	0.0001	0.00005	10.9	•	0.702	•
Skewness	**1.88**	0.43	0.10	0.16	NA		0.387	◊
Kurtosis	**10.26**	2.44	3.50	5.02	21.8	∘	0.411	∘
**Full brain**								
Mean	**0.197**	0.013	0.00003	0.0003	8.4	••	0.097	◊
Median	**0.183**	0.013	0.00001	0.0003	9.6	••	0.042	◊
Mode	**0.164**	0.020	0.00008	0.0006	14.9	•	0.120	◊
Std	**0.076**	0.010	0.00008	0.00005	9.3	••	0.613	•
Skewness	**1.37**	0.24	0.06	0.004	NA		0.938	••
Kurtosis	**6.26**	1.17	1.38	0.12	5.4	••	0.922	••

Mean and standard deviation (two first columns) of mean, median, mode, standard deviation (std), skewness and kurtosis of *α* were calculated over all data (n = 22, from 11 volunteers scanned twice). Note that the mean and std over all data were labeled mean_all_ and std_all_ to avoid confusion with the mean and std of the measurements of C_1_ and *α* for each volunteer and scan. Inter- and intra-subject variance (Inter-Var and Intra-Var respectively), coefficient of variation (CV, in %) and intra-class correlation (ICC) were calculated as described in the Statistical analysis section (see text). NA: Not available; the CV is not appropriate for measures (e.g., skewness) that are not inherently non-negative. Symbols represent subjective evaluation of CV and ICC: •• = very good (ICC ⩾ 0.8 or CV ⩽ 10%), • = good (0.6 ⩽ ICC < 0.8 or 10% < CV ⩽ 20%), ∘ = fair/moderate (0.4 ⩽ ICC < 0.6 or 20% < CV ⩽ 30%), ◊ = poor (ICC < 0.4 or CV ⩾ 30%).

### Mean_all_ and std_all_

**C_1_**—We can observe that mean C_1_ over the 22 scans had a mean_all_±std_all_ of 11.4±1.5 mM in GM and slightly higher 13.6±2 mM in WM, std was 4.5±0.7 mM in both GM and WM, skewness was 0±0.3 in both GM and WM, kurtosis was 3.4±0.5 in GM and slightly higher 4.0±1.7 in WM (but with higher variability). Median and mode values were in the same range as the mean value, in GM and WM respectively. For full brain, all the measures were of the same order of magnitude than in GM and WM.

**α**—We can observe that mean *α* measured over the 22 scans had a mean_all_±std_all_ of 0.21±0.02 in GM and lower 0.17±0.02 in WM, std was 0.08±0.01 mM in GM and slightly lower 0.06±0.01 in WM, skewness was 1.2±0.4 in GM and higher 1.9±0.4 in WM, kurtosis was 5.7±1.8 in GM and almost doubled 10.3±2.4 in WM. Due to the positive skewness of the *α* distribution, median and mode values were slightly lower than the mean values, in GM and WM respectively. For full brain, all the measures were of the same order of magnitude than in GM and WM.

### CV

**C_1_**—We can observe that the CV in GM was ∼12% for mean, median, mode and std, and lower ∼6% for kurtosis. CV values were generally higher in WM: ∼12% for mean and median, ∼19% for mode, ∼16% for std and ∼40% for kurtosis. The CV values in full brain were of the same order of the values for GM and WM, except in the case of kurtosis where CV is low ∼6%.

**α**—We can observe that CV in GM was ∼10% for mean and median and std, ∼15% for mode, and much higher ∼27% for kurtosis. The same trend occurred in WM, with CV ∼12% for mean, median and std, ∼18% for mode and ∼22% for kurtosis. The CV values in full brain were of the same order of the values for GM and WM, except in the case of kurtosis where CV was low ∼5%.

### ICC

**C_1_**—In both GM and WM, ICC was moderate ∼0.5 for mean, median and std, and good to very good ∼0.6–0.8 for mode and skewness. For kurtosis, ICC was high ∼0.8 in GM, but much lower ∼0.4 in WM, due to intra-subject variance in this tissue. For full brain, ICC was moderate ∼0.5 for mean, median, mode and std, but good to very good for skewness (∼0.7) and kurtosis (∼0.9), respectively.

**α**—In both GM and WM, ICC was low ∼0.1–0.2 for mean, median and mode, good ∼0.7 for std, and poor to moderate for skewness and kurtosis (∼0.4–0.5). In full brain however, ICC for skewness and kurtosis were both very good ∼0.9, while it was still good for std (∼0.6), and very low for mean, median and mode (∼0.1).

## Discussion

Mean, median, mode and std values of C_1_ were all in good agreement with values from the literature for intracellular sodium concentrations in healthy brain tissues, which are generally in the range 5–15 mM [20, 41]. The mean, median and mode *α* values were also in good agreement with values from the literature for extracellular volume fraction in healthy brain tissues, generally in the range 0.15–0.25 [8, 11]. From the mean skewness and kurtosis values, we found that the C_1_ distribution over whole GM, WM or full brain was very close to the normal (Gaussian) distribution (skewness = 0, kurtosis = 3), while the *α* distributions in GM and WM deviate from the normal distribution. The positive skewness (range 1–2) for *α* indicates a higher range of values above the mean value, while higher kurtosis (> 3) indicates a more ‘peaked’ distribution of *α*, with fatter tails, compared to normal distribution. These disparities for *α* from the normal distribution occur mostly because of both methodological and physiological reasons. From the methodology point-of-view, due to the low resolution of the ^23^Na and ^1^H images, both C_1_ and *α* maps exhibit residual partial volume effect that can influence their distributions, mainly in regions close the CSF which will therefore have high *α* > 0.2 (increase skewness of *α*). Moreover, incomplete inversion of the magnetization and noisy data can also generate voxels with both higher and lower values compared to normal distribution and therefore create fat tails. For the physiological point-of-view, the difference in kurtosis of *α* between GM and WM could be explained by the differences in cell packing characteristics and structure [42] between these two tissues. It is generally considered that WM has a more anisotropic structure than GM [42, 43], due to the presence of axons and glial cells, associated with a wider range of extracellular volume fractions (therefore higher kurtosis that GM), and potentially more high values of *α* > 0.2 (higher skewness than GM). As intracellular sodium concentrations in all cells in the brain don’t vary too much from the 5–15 mM range, the C_1_ distributions don’t differ significantly between GM and WM.

The skewness and kurtosis of both the C_1_ and *α* distributions measured with this protocol may also be in part the results of the discrepancy between the edge definitions of the ^1^H DIR images (2.5 mm resolution), the ^23^Na aTSC images (5 mm resolution for the data acquisition) and the ^23^Na aISC images (6.7 mm resolution). Because of all these limitations, it can be difficult with the present method to assign a relevant biological or clinical meaning to the skewness and kurtosis of the distributions. As we are more interested in changes in these variables from healthy to non-healthy tissues, and because the exact same protocol is applied to all subjects, this lack of accurate biological interpretation should not impair the ability of the method to assess changes in C_1_ and *α* due to pathologies.

Except for C_1_ kurtosis where the CV > 40%, all measures of C_1_ and *α* can be considered good variables for detecting changes over time in individuals (intra-subject variations), provided that the changes are above 20% of their mean values. From the error propagation calculated in [23], the uncertainty on the measurements of both C_1_ and *α* was calculated as around 40% for standard uncertainties on water fraction (w) and extracellular sodium concentration (C_2_) approximations. Taking CV and uncertainties into account, it is therefore reasonable to estimate that in general the proposed method would be able to detect changes in C_1_ and *α* of about 50% and above (to be on the safe side), which corresponds to mean C_1_ > 20–25 mM and mean *α* > 0.3, both for local (regions-of-interest) or global (whole tissue) measurements. Due to all the model assumptions, the low SNR of sodium MRI, the error propagation due to uncertainties in the relaxation times, in the water fraction and in other post-processing parameters, we can estimate that changes below 50% will not be a reliable. If we consider only changes of 50% and above for mean C_1_ and *α*, as expected in many neuropathologies from the literature [5–11], the combination of C_1_ and *α* measurements can also provide good classification between different groups of subjects (moderate to good ICCs), mainly if measurements are performed over full brain.

For example, in stroke, it is generally estimated that an apparent total tissue sodium concentration (aTSC) of 65–70 mM is a marker of irreversible tissue damage (cell death). This would correspond to an intracellular sodium concentration of about 40–50 mM (see for example Ref. [3, 17, 18]). We could therefore reasonably expect that 20mM < C_1_ < 50 mM would correspond to loss of homeostasis that might be reversible, and C_1_ > 50 mM would correspond to irreversible loss of cell viability. For cancer, tumor cells have altered physiology, such as evasion of apoptosis, limitless replicative potential, tissue invasion [44], and therefore also altered homeostasis. In that case, it can be expected that increases in C_1_ and *α* above 50% and even more can be linked to tumor malignancy, and potentially grading and staging (See Ref. [2, 13–15] and references within).

As ICC for skewness and kurtosis in full brain are very good, these two variables seem to be the best features for detecting differences between subjects. From the simulations of different lesions (see S1 File), we can see that compact fluid lesions (cystic-like) can be detected on the *α* maps, compact solid lesions (tumor-like) can be detected on the C_1_ maps, but random lesions cannot be detected on either *α* or C_1_ maps. However, these lesions (compact and random) can be detected on the C_1_ and *α* distributions (over whole brain), mainly through changes in skewness and kurtosis, while other measures remained almost unchanged.

As a first approximation in this study, we assume that all extracellular sodium is either completely suppressed by IR (with TI optimized to suppress fluids) or reduced within noise level (for ‘bound’ extracellular sodium, which can be expected to have intermediate T1 between fluid sodium T1 and bound sodium T1 from within the cells). Future studies will take this into account by using a more complex tissue model (more compartments) which will include the presence of sodium with restricted motion within the interstitial space, that is different from extracellular CSF or plasma space.

Due to time constraints for the subjects (we try to keep scans of the volunteers in about 45–60 min maximum) and for scanner availability, the sodium scans were optimized to last 11 min and 17 min by using shorter TRs (80 ms and 100 ms). Although these TRs are not optimized for full recovery of the longitudinal magnetization, we found that the parameters used were a good compromise between SNR and total acquisition time for this pilot study. Relaxation times in pathologies are unknown and might affect the quantification, but we expect these relaxation times within and outside pathological cells to be still quite similar to the relaxation times within and outside normal cells, respectively. For example, assuming an average T1 ∼30 ms in parenchyma [2], a variation of ±20% in T1 of the tissue will induce a variation of signal of only 3.6% in sodium images acquired without fluid suppression and TR = 80 ms, and a variation of about 5% in sodium images acquired with fluid suppression and TR = 100 ms, compared to acquisitions with TR = 150 ms (fully recovered magnetization with TR = 5×T1). These signal variations will propagate to the C_1_ and *α* quantification and are included in the general error propagation calculated in Ref. [23] for aTSC and aISC quantification, which add up to about 40% when combined with water fraction and extracellular sodium concentrations uncertainties.

Partial volume effect is a real concern for this data and was already partially discussed in Ref. [23] (p.4–5). Images were acquired with different resolutions in this pilot study for practical reasons: we wanted to be able to scan people within a reasonable time without losing too much SNR, mainly for sodium with fluid suppression. We are now working on ways to improve the SNR of the images (new multichannel coil, iterative reconstruction such as compressed sensing, data denoising) in order to increase the resolution of the images, with and without fluid suppression (we expect to reach 3–4 mm isotropic for both, with about 10–12 min acquisition time for each acquisition).

No sodium signal from zero concentration in reference phantoms was measured for the linear regression, as it is hidden in the noise of the images. As sodium images have low SNR, we observed that adding measurements of [Na] = 0 mM (therefore noise) can induce large errors in the quantifications, which are very sensitive to the slopes of the linear regressions. Future increases of the SNR of the images, as already described above, will improve the robustness of the method to noise.

In conclusion, estimating the pseudo-intracellular sodium concentration (C_1_) and pseudo-extracellular volume fraction (*α*) in brain in vivo is feasible using a combination of DIR proton MRI and sodium MRI, with moderate to good repeatability (ICC) and CV. This preliminary method will be furthermore improved in future studies by implementation of compressed sensing reconstruction [45] and data denoising methods [46] for increasing the signal-to-noise ratio, accelerating sodium acquisitions, and/or increasing the resolution of the sodium images. Water fraction estimation with proton MRI [37, 38], which was considered as fixed in the present work, will also be included in the future, as this parameter also changes with pathologies. Future studies with multichannel RF coils will also include the calculation of sodium B_1_ maps and the development of B_1_ inhomogeneities correction for the calculation of C_1_ and *α*, in combination with increased SNR and resolution, to reduce CSF partial volume effect and inefficient CSF suppression effect on data quantification. Next steps will include estimating the efficiency of the method on patients for assessing different neuropathologies such as multiple sclerosis, brain tumors, traumatic brain injuries, or Alzheimer’s disease, either locally on C_1_ and/or *α* maps, or globally using the distributions of C_1_ and/or *α* over whole GM, whole WM or whole brain.

## Supporting Information

S1 Dataset Dataset of C_1_ and *α* measurements (mean, median, mode, standard deviation, skewness, kurtosis) in GM, WM and full brain, for all scans of all volunteers. (XLSX)

S1 File **Figure A.** Pseudo-intracellular sodium concentration (C_1_) maps of the brain of a healthy volunteer (1 axial slice) with artificial ‘fluid’ and ‘solid’ inclusions.
**Figure B.** Distributions of all pseudo-intracellular sodium concentration (C_1_) values in full brain (GM+WM, black), GM (blue), WM (red) from a volunteer, with artificial ‘fluid’ and ‘solid’ inclusions.
**Figure C.** Pseudo-extracellular volume fraction (*α*) maps of the brain of a healthy volunteer (1 axial slice) with artificial ‘fluid’ and ‘solid’ inclusions.
**Figure D.** Distributions of all pseudo-extracellular volume fraction (*α*) values in full brain (GM+WM, black), GM (blue), WM (red) from a volunteer, with artificial ‘fluid’ and ‘solid’ inclusions.
(PDF)
