# Supplementary material for: Repeatability of Quantitative Sodium Magnetic Resonance Imaging for Estimating Pseudo-Intracellular Sodium Concentration and Pseudo-Extracellular Volume Fraction in Brain at 3 T
Source: PLoS One. 2015 Mar 9;10(3):e0118692. doi: 10.1371/journal.pone.0118692 (PMC4353709; doi:10.1371/journal.pone.0118692)
Supplement: S1 File — Figure A. Pseudo-intracellular sodium concentration (C1) maps of the brain of a healthy volunteer (1 axial slice) with artificial ‘fluid’ and ‘solid’ inclusions. Figure B. Distributions of all pseudo-intracellular sodium concentration (C1) values in full brain (GM+WM, black), GM (blue), WM (red) from a volunteer, with artificial ‘fluid’ and ‘solid’ inclusions. Figure C. Pseudo-extracellular volume fraction (α) maps of the brain of a healthy volunteer (1 axial slice) with artificial ‘fluid’ and ‘solid’ inclusions. Figure D. Distributions of all pseudo-extracellular volume fraction (α) values in full brain (GM+WM, black), GM (blue), WM (red) from a volunteer, with artificial ‘fluid’ and ‘solid’ inclusions. (PDF) [file pone.0118692.s002.pdf]

Supporting Information

Repeatability of quantitative sodium magnetic resonance imaging for estimating pseudo-intracellular sodium concentration and pseudo-extracellular volume fraction in brain at 3 T

Guillaume Madelin[1,*], James Babb[1], Ding Xia[1], Ravinder R. Regatte[1]

[1] Center for Biomedical Imaging, Department of Radiology, New York University Langone Medical Center, New York, NY 10016, USA

[*] E-mail: guillaume.madelin@nyumc.org

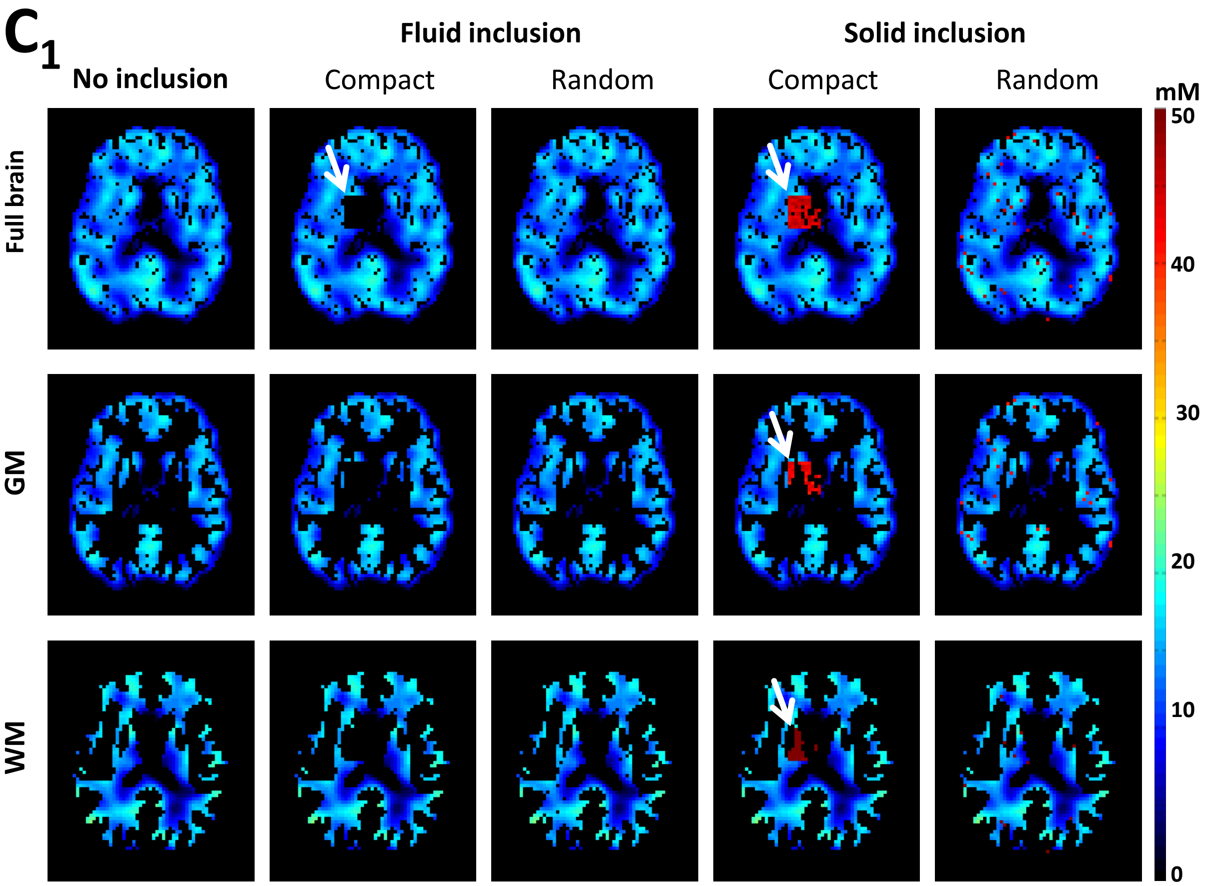

Figure A. Pseudo-intracellular sodium concentration (C_1) maps of the brain of a healthy volunteer (1 axial slice) with artificial 'fluid' and 'solid' inclusions. These inclusions were generated artificially on the aTSC and aISC maps before α and C_1 quantification processing. Artificial 'fluid' inclusion were generated using aTSC = 120 mM and aISC = 5 mM (simulating fluid cyst or other effusions) and 'solid' inclusions were generated with aTSC = 55 mM and aISC = 25 mM (simulating tumors or dying cells). See Ref. [1] for more details. Inclusions were either compact ($10 \times 10 \times 10$ voxels) or random (1000 voxels distributed randomly within full brain), in order to simulate more localized or more diffuse lesions, respectively. The white arrow indicates the position of the compact inclusion (mostly in GM, with small overlap on WM). Random noise in the range [-2,2] mM was also added to the aISC and aTSC values of the inclusions. Note the excellent spatial detection of the compact 'solid' inclusion ($C_1 \sim$45-50mM) and the loss of signal due to 'fluid' inclusions (which have almost no intracellular sodium concentration). Random inclusions remained practically undetectable (a few voxels from 'solid' inclusions with high C_1 values can be detected with a close look). Abbreviations: GM = grey matter, WM = white matter, full brain = WM+GM.

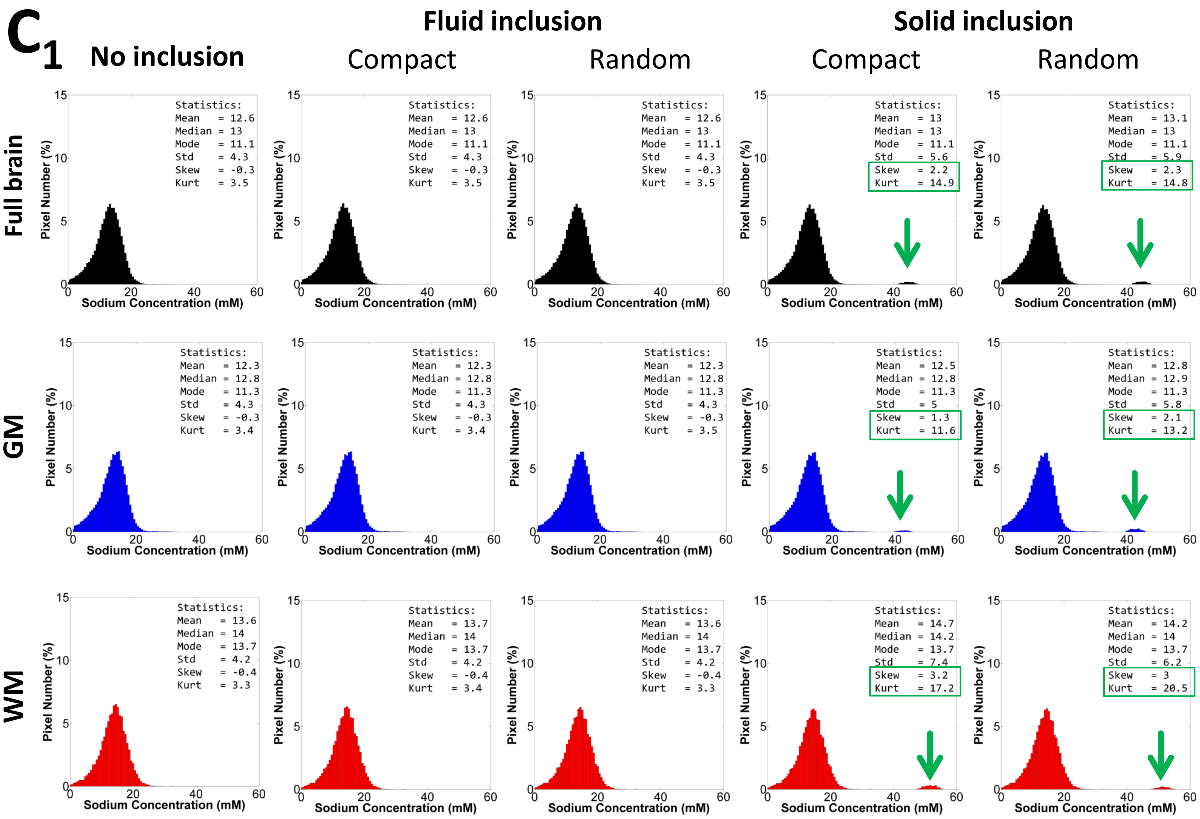

Figure B. Distributions of all pseudo-intracellular sodium concentration (C_1) values in full brain (GM+WM, black), GM (blue), WM (red) from a volunteer, with artificial 'fluid' and 'solid' inclusions. See caption of Fig. S1 for details. Statistical parameters of the distributions are included in the top right corner of each histogram. Pixel number is given in % of the total number of pixels from the whole 3D data in full brain, in GM and in WM, respectively. Note the localized new distribution of C_1 values (indicated by green arrows) in the range 40-55 mM due to the presence of the 'solid' inclusion, either compact (which can be detected on C_1 maps) or random (not detected on C_1 maps). This inclusion represent about 1.15% of the total brain volume (1000 voxels over 86571 voxels in whole GM+WM). The mean, median, mode or std of the full C_1 distributions are almost unchanged compared to the same data without inclusion, but the skewness and kurtosis are significantly increased by a factor of around 5-6 and 3-6, respectively, compared to values swithout inclusions (this factor was calculated using absolute values for skewness). These two later parameters could therefore allow the detection of small changes (of the order of a few %) in the global distributions of C_1 values within the brain, due to either localized or diffuse 'solid' lesions.

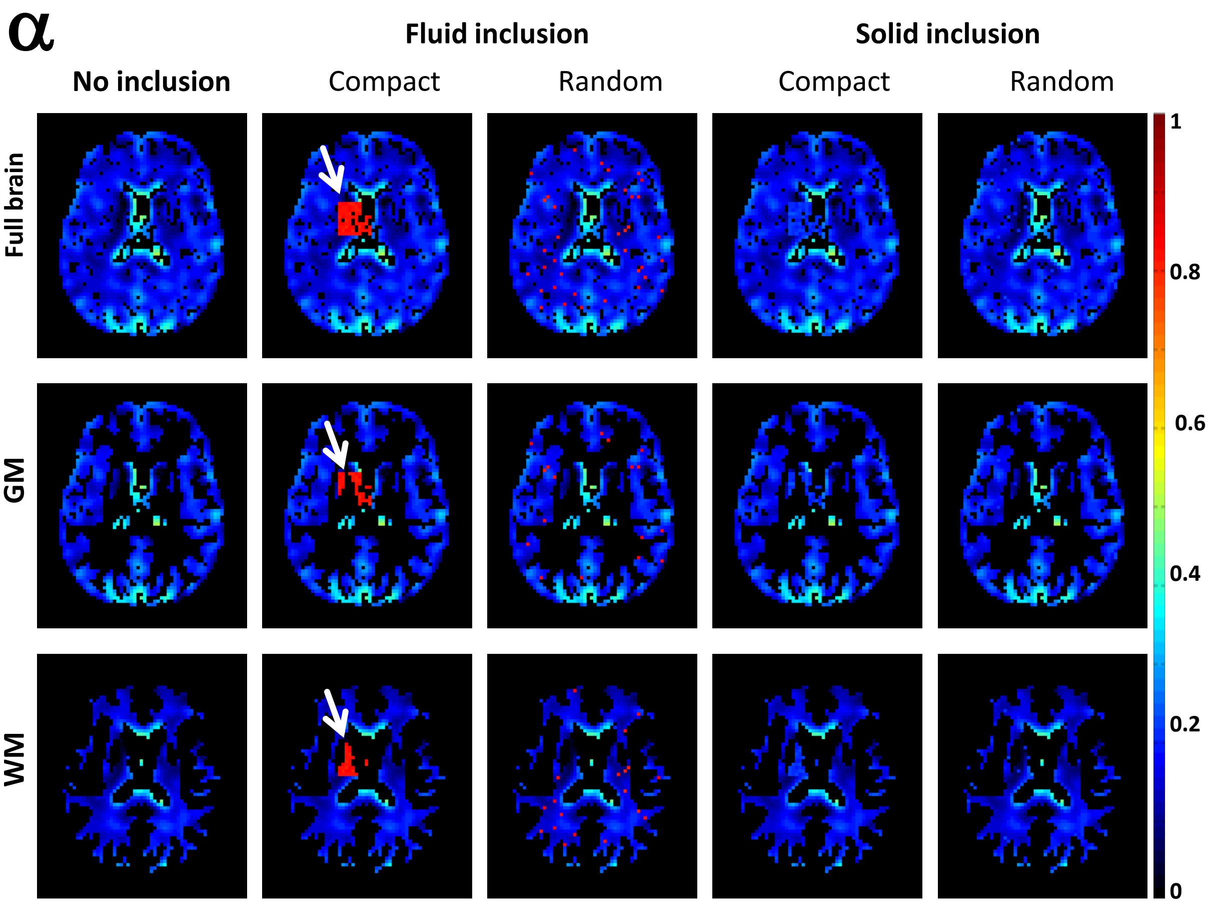

Figure C. Pseudo-extracellular volume fraction (α) maps of the brain of a healthy volunteer (1 axial slice) with artificial 'fluid' and 'solid' inclusions. These inclusions were generated artificially on the aTSC and aISC maps before α and C_1 quantification processing. Artificial 'fluid' inclusion were generated using aTSC = 120 mM and aISC = 5 mM (simulating fluid cyst or other effusions) and 'solid' inclusions were generated with aTSC = 55 mM and aISC = 25 mM (simulating tumors or dying cells). See Ref. [1] for more details. Inclusions were either compact (10×10×10 voxels) or random (1000 voxels distributed randomly within full brain), in order to simulate more localized or more diffuse lesions, respectively. The white arrow indicates the position of the compact inclusion (mostly in GM, with small overlap on WM). Random noise in the range [-2,2] mM was also added to the aISC and aTSC values of the inclusions. Note the excellent spatial detection of the compact 'fluid' inclusion (α ∼0.8) and the lack of detection of the 'solid' inclusions, either compact or random (there is an increase of intracellular sodium content in solid inclusions while keeping the extracellular volume fraction almost constant). Random inclusions remained practically undetectable (a few voxels from 'fluid' inclusions with high α values can be detected with a close look). Abbreviations: GM = grey matter, WM = white matter, full brain = WM+GM.

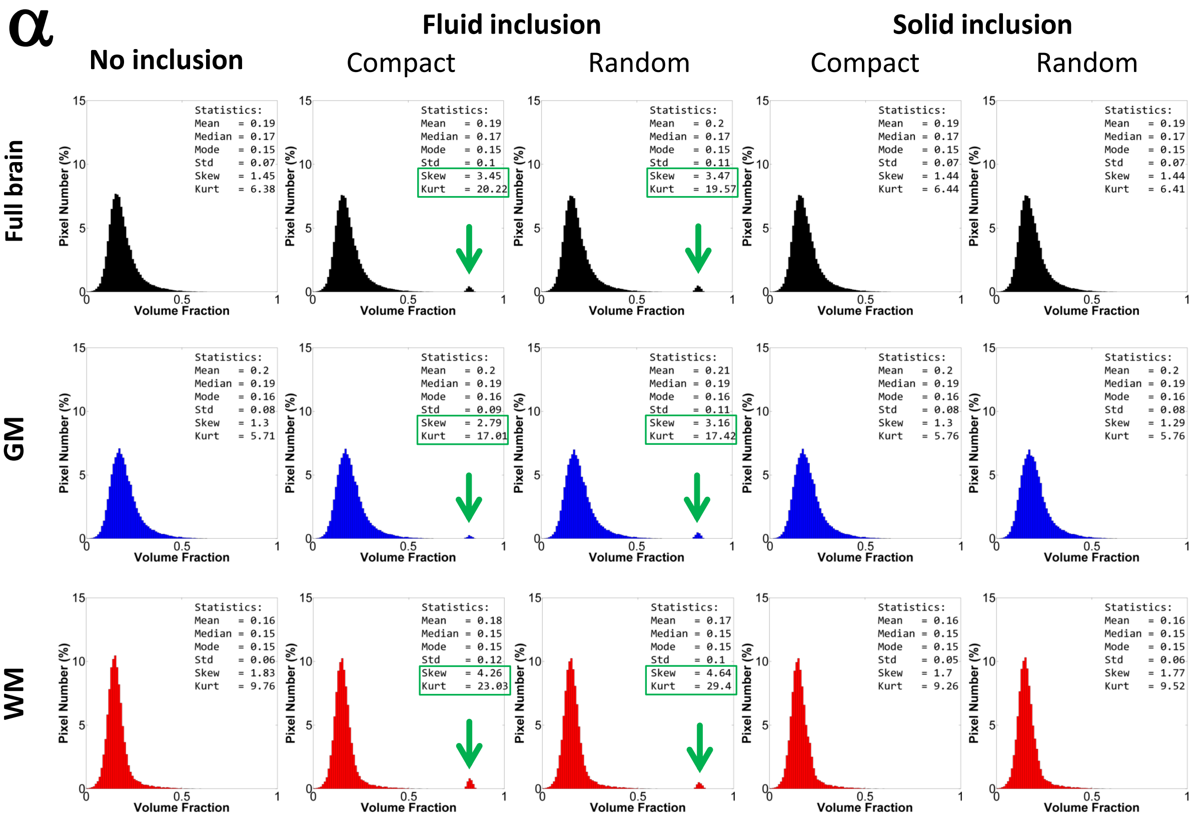

Figure D. Distributions of all pseudo-extracellular volume fraction (α) values in full brain (GM+WM, black), GM (blue), WM (red) from a volunteer, with artificial 'fluid' and 'solid' inclusions. See caption of Fig. S3 for details. Statistical parameters of the distributions are included in the top right corner of each histogram. Pixel number is given in % of the total number of pixels from the whole 3D data in full brain, in GM and in WM, respectively. Note the localized new distribution of α values (indicated by green arrows) around 0.8 due to the presence of the 'fluid' inclusion, either compact (which can be detected on α maps) or random (not detected on α maps). This inclusion represent about 1.15% of the total brain volume (1000 voxels over 86571 voxels in whole GM+WM). The mean, median, mode or std of the full α distributions are almost unchanged compared to the same data without inclusion, but the skewness and kurtosis are significantly increased by factors of around 2-3 and 3, respectively, compared to values without inclusions. These two later parameters could therefore allow the detection of small changes (of the order of a few %) in the global distributions of α values within the brain, due to either localized or diffuse 'fluid' lesions.

References

[1] G Madelin, R Kline, R Walvick, and RR Regatte. A method for estimating intracellular sodium concentration and extracellular volume fraction in brain in vivo using sodium magnetic resonance imaging. *Scientific Reports*, 4:4763, 2014. doi:10.1038/srep04763.
